# Diabetes, Diet-Health Behavior, and Obesity

**DOI:** 10.3389/fendo.2015.00033

**Published:** 2015-03-16

**Authors:** Sven Anders, Christiane Schroeter

**Affiliations:** ^1^Department of Resource Economics and Environmental Sociology, University of Alberta, Edmonton, AB, Canada; ^2^Department of Agribusiness, California Polytechnic State University, San Luis Obispo, CA, USA

**Keywords:** diabetes management, healthy eating index (2010), nutrition supplement intake, obesity, diet quality

## Abstract

High-quality diets play an important role in diabetes prevention. Appropriate dietary adherence can improve insulin sensitivity and glycemic control, and thus contribute to lifestyle improvement. However, previous research suggests that dietary adherence is arguably among the most difficult cornerstones of diabetes management. The objectives of this study are (1) to estimate whether and to what extent individuals diagnosed with diabetes show significant differences in diet quality [healthy eating index (HEI)] compared to healthy individuals, (2) to quantify whether and to what extent diabetics experience significantly higher outcomes of body mass index (BMI), and (3) to estimate whether and to what extent dietary supplementation impacts diabetes patient’s diet quality and/or BMI outcomes. We use data from the 2007–2008 U.S. National Health and Nutrition Examination Survey (NHANES). The NHANES is the primary, randomized, and nationally representative survey used to assess the health and nutritional status in the U.S. We apply propensity score matching (PSM) to account for selection bias and endogeneity between self-reported diet and health behavir (treatment) and BMI outcomes. We control for an individual’s BMI as to capture the impact of past dietary behavior in its impact on HEI. Matching results suggest that regular dietary supplement consumption is associated with significant lower BMI outcomes of almost 1 kg/m^2^. The close relationship between diabetes and obesity has been at the center of the diet-health policy debate across Canada and the U.S. Knowledge about this linkage may help to improve the understanding of the factors that impact dietary choices and their overall health outcomes, which may lead to a more efficient and effective promotion of dietary guidelines, healthy food choices, and targeted consumer health and lifestyle policies.

## Introduction

Diabetes a major cause of mortality globally, and it has been estimated that 400 million people worldwide will suffer from it by 2030 (1). Even though genetics appears to play a crucial role in the development of diabetes, research suggests that dietary choices driven by environmental and economic factors are of crucial importance (2–6). High-quality diets play an important role in diabetes prevention. Appropriate dietary adherence can improve insulin sensitivity and glycemic control, and thus contribute to lifestyle improvement and overall quality of life (7, 8). However, previous research suggests that dietary adherence is arguably among the most difficult cornerstones of diabetes management (7, 9).

Recently, increased policy attention has been placed on efforts to improve dietary habits in order to reduce health care costs (10). The USDA developed the healthy eating index (HEI) which categorizes diet quality according to the recommendations of the 2010 Dietary Guidelines for Americans (10). The HEI is used to monitor the quality of American diets and to examine the relationship between food intake and diet-related outcomes (11). Scores are assigned based on a density approach – that is the standards for maximum scores are given as the amount of the food or nutrient per 1,000 calories. Higher HEI scores indicate closer adherence to current dietary guidelines for individual food and nutrient groups. For the adequacy components such as vegetables and fruit, a higher score indicates higher consumption. Dietary recommendations are based on the beneficial effects of consuming fruits and vegetables and explicitly emphasize their positive effects of reducing obesity and certain types of cancers (12–14). The last three components of the HEI include refined grains, sodium, and empty calories (calories from solid fats, alcohol, and added sugars) and a higher score indicates lower consumption (11, 14).

Previous studies measured the total HEI of an average U.S. consumer at 52 out of 100, with individual component HEI scores such as fruits and vegetables sub scores at about 40% of their recommended levels (15). Overall, lifestyle changes have contributed to consumers’ more favorable attitudes toward convenience items such as nutritional supplements as a perceived alternative to improving diet quality instead of consuming fruits and vegetables (16). The Canadian Diabetes Association (CDA) does not recommend a routine supplementation of diets for people with diabetes. However, studies suggest that dietary supplement manufacturers may actually encourage consumers to substitute their physician-prescribed medications with supplements. Thus, at-risk populations such as diabetics may be more prone to consuming dietary supplements, given that consumers may not be able to differentiate between technical descriptions and marketing language (17).

In the context of diabetes, the economic affordability (e.g., food security), accessibility, and acceptability (e.g., food culture) have been discussed as potential barriers to meeting and adherence to recommended dietary guidelines (18–20). The diet-health behavior of diabetes patients and strategies to overcome potential barriers to adherence to recommended dietary guidelines are key public health and diabetes health concern (21). Thus, there is need to quantify the relationship between diet quality, obesity, and diabetes.

The objectives of this study are (1) to estimate whether and to what extent individuals diagnosed with diabetes show significant differences in diet quality (HEI) compared to healthy individuals, (2) to quantify whether and to what extent diabetics experience significantly higher outcomes of body mass index (BMI), and (3) to estimate whether and to what extent dietary supplementation impacts diabetes patient’s diet quality and/or BMI outcomes. We apply propensity score matching (PSM) to quantify the possible link between diabetes status, diet-health behavior, and health outcome, as represented by individual’s HEI and obesity status. In this analysis, we control for individual’s BMI as to capture the impact of past dietary behavior in its impact on HEI.

To the best of our knowledge, no previous study has incorporated such a wide range of lifestyle, diet-health, and food culture variables when determining the relationship of diet quality and obesity in the context of diabetes. The close relationship between diabetes and obesity has been at the center of the diet-health policy debate across Canada and the U.S. (22). Knowledge about this linkage may help to improve the understanding of the factors that impact dietary choices and their overall health outcomes, which may lead to a more efficient and effective promotion of dietary guidelines, healthy food choices, and targeted consumer health and lifestyle policies.

## Materials and Methods

### Data

The analysis employs data from the 2007–2008 U.S. National Health and Nutrition Examination Survey (U.S. NHANES). The NHANES is the primary, randomized, and nationally representative survey used to assess the health and nutritional status in the U.S. Data from the various NHANES survey cycles have been used in a number of economic studies focused on individual health behavior, consumption choices, and other related issues (15, 23–27).

For the analysis in this paper, we select 8,273 adult NHANES respondents aged 20 and older, with specific emphasis on their diabetes status. From the large pool of information elicited through NHANES variables, we identify and select: health status, diet quality, lifestyle, food culture, food security, and demographic information. Table 1 summarizes the descriptive statistics for the variables available for analysis.

**Table 1:** **Descriptive statistics of model variables**.

Variable	Description	Mean	SD
**Health status**			
Body mass index	Weight (kg)/height (m^2^)	28.97	6.67
Diabetes	=1 if respondent has been diagnosed with diabetes by doctor or health professional	0.13	0.33
Blood pressure	=1 if respondent has been told by doctor or health professional to have high blood pressure	0.96	0.21
**Diet quality (healthy eating index 2010)**			
HEI total	Total diet quality/1,000 calories (or as % of calories), sum of 12 components (9 adequacy, 3 moderation), max 100 points	54.60	1.09
HEI total vegetables	≥1.1 cup equiv./1,000 kcal, includes any beans and peas not counted as total protein foods, max 5 points	3.15	0.08
HEI greens & beans	≥0.2 cup equiv./1,000 kcal, includes any beans and peas not counted as total protein foods, max 5 points	2.19	0.19
HEI total fruit	≥0.8 cup equiv./1,000 kcal, includes 100% fruit juice, max. 5 points	3.07	0.14
HEI whole fruit	≥0.4 cup equiv./1,000 kcal, includes all forms except juice, max 5 points	4.10	0.22
HEI whole grains	≥1.5 oz. equiv./1,000 kcal, max 10 points	1.95	0.09
HEI dairy	≥1.3 cup equiv./1,000 kcal, includes all milk products, such as fluid milk, yogurt, cheese, and fortified soy beverages, max 10 points	5.58	0.15
HEI total protein foods	≥2.5 oz. equiv./1,000 kcal, beans and peas included (and not with vegetables), max 5 points	5.00	0.00
HEI seafood & plant protein	≥0.8 oz. equiv./1,000 kcal, includes seafood, nuts, seeds, soy products (other than beverages), max 5 points	2.99	0.13
HEI fatty acids	>2.5, ratio of poly- and monounsaturated fatty acids to saturated fatty acids, max 10 points	3.96	0.11
HEI sodium	≤1.1 g/1,000 kcal, max 10 points	4.35	0.13
HEI refined grains	≤1.8 oz. equiv./1,000 kcal, max 10 points	6.78	0.13
HEI empty calories	≤19% of energy, calories from solid fats, alcohol, and added sugars; threshold for counting alcohol >13 g/1,000 kcal, max 20 points	11.49	0.35
**Lifestyle**			
Very active	=1 if respondent’s self-rated daily activity is very vigorous	0.19	0.41
Drinker	=1 if respondent had at least 12 alcohol drinks in a year?	0.71	0.51
Smoker	=1 if respondent has smoked at least 100 cigarettes in entire life and is currently smoking	0.48	0.56
Supplement	=1 if respondent has taken nutrition supplements in past 30 days	0.46	0.50
**Food culture**			
White	=1 if respondent is non-Hispanic white	0.47	0.50
Black	=1 if respondent is non-Hispanic Black	0.21	0.41
Hispanic	=1 if respondent is Hispanic	0.11	0.32
Other race	=1 if respondent is of other race	0.21	0.41
Citizen	=1 if respondent is a U.S. citizen	0.69	0.34
Household size	=total number of people living in household	3.13	1.66
**Food security**			
Food stamps	=1 if respondent has ever received food-stamps	0.24	0.48
Food bank	=1 if respondent has received food from food bank	0.08	0.36
**Demographics**			
Male	=1 if respondent is male	0.49	0.50
Age	=age of respondent in years	50.37	17.80
High school	=1 if respondent went to high school	0.25	0.43
Some college	=1 if respondent went to some college	0.26	0.44
Graduate	=1 if respondent graduated from college and above	0.19	0.39
Household Inc., 1	=1 if annual HH income is between $0–$24,999	0.35	0.48
Household Inc., 2	=1 if annual HH income is between $25,000–$49,999	0.22	0.42
Household Inc., 3	=1 if annual HH income is between $50,000–$74,999	0.19	0.39
Household Inc., 4	=1 if annual HH income is between $75,000–$99,999	0.09	0.28
Household Inc., 5	=1 if annual HH income is $100,000 and over	0.12	0.33
Married	=1 if respondent is married/common law	0.60	0.49
Divorced	=1 if respondent is divorced or separated	0.23	0.42

*Source: calculations based on U.S. NAHENS data, 2007/08 cycle (*n* = 8,273)*.

The health status variables serve a marker for the individual’s current health status, based on the fact that longitudinal studies directly link poor diet quality to deteriorating health indicators such as obesity, diabetes, and overall physical health, which in turn are indicators of a higher risk of cardio-vascular disease (28). We employ the BMI to capture the impact of past eating behavior on HEI. Obesity has been at the center of the diet-health policy debate in the United States, and the focus of a growing number of economic studies [e.g., Ref. (22, 29)]. In our data set, respondents have an average BMI of 29, which is at the top range of the overweight range (25 ≤ BMI < 30), which means it is getting close to the obesity category (BMI ≥ 30). In addition, we use binary variables to reflect whether an individual has been told by a health professional that their blood pressure is high or that the individual has diabetes. Table 1 shows that 13% of respondents have been diagnosed with diabetes and 96% have been told that they have high blood pressure.

Based on detailed NHANES dietary recall data consisting of two 24-h multi-pass dietary recall interviews, we computed HEI 2010 component scores and total HEI scores as the main measure of diet quality for all NHANES respondents in this analysis using the approach described in Kahle and Buckman (30). The first dietary recall interview is collected in person, while the second run is collected via telephone within 10 days of the first. NHANES interviewers record the amount of food actually consumed, rather than the amount of food that is purchased, which allows for a more precise measurement of food intake. In addition, NHANES survey interview questionnaires aim at collecting supplemental consumption, lifestyle, and demographic characteristics (31).

We computed individual HEI component scores that add to a maximum total score are computed using the MyPyramid Equivalents Database (MPED) and NHANES 07-08 datasets to develop a scoring system for an optimal diet quality (HEI = 100). Each dietary category (e.g., total fruit intake) scores between 0 and a maximum (score) contribution to the overall diet quality as expressed in the HEI. For instance, no intake of fruits leads to a score of zero, whereas the contribution of a high level of fruit consumption to diet quality is capped at maximum 5 points of any total HEI score. The average total HEI is 54.60, which is comparable to the findings in other studies (15). Previous studies directly link poor diet quality to deteriorating health indicators such as diabetes, obesity, cholesterol levels, and overall physical health (32).

Lifestyle indicators include health and risk behaviors such as exercise frequency, frequent alcohol consumption, smoking, and the intake of nutrition supplement. These lifestyle factors may significantly influence an individual’s health status and food choice behavior (33). In our sample, we selected participants who indicated performing very vigorous daily activities, given that increased sedentary time may also be a proxy for consuming unhealthier food or snack choices (33). On average, 19% of the respondents in our sample indicate that they perform very vigorous activities (see Table 1).

The frequency of alcohol consumption and nicotine, an appetite suppressant, can be understood as health indicators, markers of an individual’s current health status. The Dietary Guidelines for Americans 2010 recommends that women consume no more than one alcoholic drink per day, and men no more than two. The majority of our respondents, 71%, report that they consume at least 12 alcohol drinks per year. Huston and Finke (34) suggest that smokers tend to have high discount rates when it comes to instant gratification versus their personal future health and longevity. Smokers also have been shown to have lower levels of diet quality (35). About half (48%) of the respondents in our sample are smokers.

Given the available definition in NHANES 2007–2008, we measured vitamin supplement intake as a binary variable, which indicates whether the respondent took any vitamins, minerals, or dietary supplements during the past month. Table 1 shows that about half (46%) of our sample reports to take nutrition supplements.

According to a recent study by Schroeter, Anders, and Carlson (15), diet quality is strongly interrelated with food culture. Our sample consists of 47% Non-Hispanic Whites, 21% Non-Hispanic Blacks, and 11% Hispanics. Furthermore, eating habits formed during childhood have been shown to have a lasting impact on adult food habits (36). As such, food culture includes factors such as heritage or ethnicity. In our sample, about 31% of respondents are immigrants. We included household size to capture differences in food culture at home, as larger households may be more likely to cook more often than smaller size or single households. Table 1 shows that the average household consists of three members. The latter can serve as proxies for the types of foods and/or traditional consumption patterns an individual has been exposed to over a long period of time (35).

We consider two variables to represent food security, i.e., whether respondents receive food stamps or food from the food bank. As Table 1 indicates, about a quarter of the sample (24%) are food stamp recipients and 8% receive food from the food bank.

Several demographic variables may impact individual’s diet-health behavior, such as gender, age, educational attainment, household income, or marital status. Previous studies have shown that improved dietary choices – frequent consumption of fruits and vegetables – are typically less prevalent among men [e.g., Ref. (34, 35)]. About half (49%) of our sample is male and the sample average age is 50.4 years (see Table 1). With increasing age, people tend to eat a diet of higher quality that contains less energy, since the benefits of health and good nutrition may become more apparent (22). Education, a proxy for knowledge, information, and awareness of healthy lifestyle practices, and willingness to invest in long term health (37) may result in overall higher diet quality and HEI.

In addition to age, gender, and education, we classify respondents into five income groups to capture the association between income and diet quality emphasized by previous economic analyses of diet and health (38–40). The largest income category is the lowest income group, which means that 35% of the respondents are earning up to $24,999. Moreover, Jeffrey and Rick (41) found marriage to be associated with higher consumption of calorie-dense foods and lower frequency of exercise. The majority (60%) of our sample is married, while 23% are divorced or separated.

### Model

We build the theoretical foundation of the analysis on Becker’s model on investment in human capital and Grossman’s seminal work on health capital. Grossman described and formalized the process by which people are endowed with a certain stock of health, which deteriorates over a person’s life time (42). How fast a person’s health status deteriorates depends, among other things, on investments made in “good health” through certain health behaviors. “Good health” can be maintained through a variety of ways including nutrition, medical care, and other relevant lifestyle choices.

The empirical analyses of individual’s diet-or health behavior in the context of specific health outcomes is typically complicated by potential two problems. The first problem is endogeneity between key variables of interest. The second issue involves the measurement error resulting from self-selection bias, a problem often encountered in consumer survey studies. In such circumstances, the use of simple regressions analysis may lead to biased and unreliable empirical results (43). A common econometric solution to such problems is the use of instrumental variable estimators (IV). However, in the context of studies in the areas of food, diet, and health behavior, it is often difficult, if not impossible, to find suitable instruments that can correct the inherent biases in the underlying data (29). Therefore, we choose PSM to account for the possible endogeneity of diet quality, diabetes status and obesity outcome (BMI), and the potential selection bias in the self-reported dietary data.

The rationale behind PSM, originally developed by Rosenbaum and Rubin (44), is to estimate treatment effects in the context of interventions (medical, policy, or otherwise) when standard randomized control trail methods aren’t feasible (45). In health economics and fields of food consumption studies, PSM methods have been employed to analyze how consumers that were exposed to a particular treatment (e.g., food label usage) differed from those who reportedly did not receive the same treatment (3, 46, 47). As such, PSM, it is a widely used method in health-related fields.

In this study, NHANES respondents who reported to have been diagnosed with diabetes by their doctor or health professional are classified as the treatment group, with all other respondents representing the control group (healthy individuals). The propensity score function or treatment selection model describes the conditional probability of having been diagnosed with diabetes:

(1) $$\begin{array}{llll} \text{Diabetes} & =\text{f(blood pressure, HEI total, very active, drinker,}\; & & \;\\ & \;\;\text{smoker, supplement, black, hispanic, other race,}\; & & \;\\ & \;\;\text{citizen, household size, food stamps, food bank,}\; & & \;\\ & \;\;\text{male, age, high school, some college, graduate,}\; & & \;\\ & \;\;\text{household income 5, married),}\; \end{array}$$

where ‘Diabetes’ is the binary dependent variable. We estimate the propensity score function (1) with the main purpose of balancing the characteristics of respondents in the treatment and control groups. The rational behind balancing is to test and assure that observations with the same propensity score of interest do also have the same distribution of observable characteristics as expressed in the covariates in the above score function. After balancing out the characteristics between diabetes patients and healthy individuals, the comparison of propensity scores for (1) BMI outcomes and (2) diet quality (HEI) across both groups can be conducted in an unbiased fashion. We estimate the average effect of having been diagnosed with diabetes on the two diet-health outcomes of interest (BMI and HEI), using different matching algorithms established in the literature: Nearest Neighbor, Caliper (Radius), Stratification and Kernel matching (48).

## Results

We find that the relationship between diabetes, diet quality, and BMI is not a causal one. However, a positive diagnosis for diabetes may have an effect on an individual’s diet-health behavior and overall diet quality, which may in turn have an effect on a relevant diet-health outcome such as the BMI. We therefore hypothesize that diabetes patients on average will have a higher HEI score and lower BMI score than their healthy counterparts. Table 2 summarizes the factors and characteristics associated with selection into the treatment group of diabetes patients.

**Table 2:** **Propensity score function, diabetes**.

Variables	Coefficient	SE
Constant	0.708	2.24
**Health status**
Blood pressure	0.048	0.35
**Diet quality (healthy eating index 2010)**
HEI total	− 0.059	0.04
**Lifestyle**
Very active	− 0.987^c^	0.18
Drinker	− 0.343^c^	0.09
Smoker	0.060	0.08
Supplement	0.107	0.26
**Food culture**
Black	0.728^c^	0.11
Hispanic	0.278^a^	0.16
Other race	0.335^b^	0.14
Citizen	0.467^c^	0.18
Household size	− 0.059^a^	0.03
**Food security**
Food stamps	0.105	0.11
Food bank	0.002	0.13
**Demographics**
Male	0.209^b^	0.10
Age	0.981^c^	0.10
High school	− 0.402^c^	0.12
Some college	− 0.397^c^	0.12
Graduate	− 0.789^c^	0.16
Household Inc., 5	− 0.075	0.17
Married	0.052	0.10
Observations, *n* = 5,064	Pseudo *R*^2^ = 0.981
Log-likelihood = −1,713.75	

*^a–c^Indicate significance at the 99, 95, and 90% level*.

As Table 2 shows, all signs are as predicted. Our best performing treatment selection model, which is focused on balancing the characteristics of diabetes patients with those of healthy individuals, does provide insights into those characteristics that are predictors of individual’s diabetes status.

We model health indicators based on respondent’s reported health status, rather than NHANES’ medical exam results. We assume that with an individual’s unawareness about her/his own health status, diet behavior would not be changed to counteract the condition. However, the results show that past diet behavior and health status (e.g., high blood pressure) are not significant identifiers of diabetes status. Health experts continue to emphasize the importance of regular health-enhancing activities, including the consumption of a well-balanced diet and physical activity (32, 36). It is plausible to assume that time spent exercising may be positively correlated with eating a healthy diet. Lifestyle factors are among the key variables explaining individual’s probability of being diagnosed with diabetes, such as leading an active lifestyle, which exhibits the largest effect in our model. Drinking alcohol negatively affects the probability of being diagnosed with diabetes.

Food culture in relation to ethnic heritage seems to play an important role in determining diabetes status. A well-documented case in point emphasizing the interplay of diet quality, up-bringing, and ethnicity is the “Hispanic Health Paradox”. The paradox suggests that U.S. immigrant’s heritage food culture may act as a protective barrier against a rapid assimilation of dietary habits. This may lead to health outcomes that are equal to or better than those of non-immigrants, despite higher poverty rates, lower education, and worse access to health care among many Hispanic immigrant groups living in the U.S. (39, 40). Being part of ethnic groups other than white is a strong predictor for diabetes status. Moreover, individuals who have resided in the U.S. for a long period of time will likely have experienced a shift in food culture toward U.S. lifestyles and dietary patterns. Thus, citizenship increases the probability of diabetes diagnosis.

Table 3 below summarizes the results of the different PSM algorithms for the comparison of diabetes patients and healthy in terms of the two outcome variables of particular interest to diet-health professionals and diabetes patients alike: diet quality and BMI.

**Table 3:** **Relationship between diabetes status, BMI, and diet quality**.

Matching algorithm	Coefficient	SE
**Total diet quality**
Nearest neighbor	0.049	0.04
Radius matching (*r* = 0.1)	0.048	0.03
Radius matching (*r* = 0.001)	0.053	0.04
Kernel	0.050	0.03
Stratification	0.051	0.04
**BMI**
Nearest neighbor	−0.546^c^	0.24
Radius matching (*r* = 0.1)	−0.670^c^	0.18
Radius matching (*r* = 0.001)	−0.399^c^	0.18
Kernel	−0.700^c^	0.17
Stratification	−0.551^c^	0.18

*^a–c^Indicate significance at the 99, 95, and 90% level. A detailed description of the matching algorithms and their implementation in STATA following Becker and Ichino (45) can be found at www.stata-journal.com/sjpdf.html?articlenum=st0026*.

The results clearly suggest that a diagnosis with diabetes is associated with significant improvements in patients’ BMI status. However, the diabetes diagnosis does not necessarily lead to a measurable improvements diet quality, as measured by the HEI-2010, when directly comparing the groups of diabetes patients with their healthy counterparts. Across matching algorithms, diabetes patients have a BMI of roughly 0.57 kg/m^2^ below the average BMI of the healthy control group of 28.5. The narrow range in estimated BMI outcomes across matching algorithms signals robustness, which stands in contrast to comparable studies that have largely reported inconclusive results (3). Moreover, more detailed analyses with different HEI component scores (available from the authors upon request) did reveal that diabetes patient’s diet did not differ from those of healthy survey participants in any of the 12 HEI categories listed in Table 1.

To further emphasize the potential impact of diabetes patient’s conscious diet-health behaviors on the important outcome variables, diet quality and BMI, we use diabetes patients’ decision to frequently consume nutrition supplements. In light of dwindling levels of fresh fruits and vegetables consumption among North American consumers and trend toward the intake of nutrition supplements, we hypothesize that diabetes patients who take nutrition supplements, thus making an effort to actively improve the overall quality of their diet, will have higher HEI scores and lower BMI scores than their non-supplement-taking peers. In a similar fashion to the previous matching exercise, we use nutrition supplement intake as a treatment selection criterion within the group of diabetes patients (Table 4).

**Table 4:** **Relationship between supplement intake, BMI, and diet quality among diabetes patients**.

Matching algorithm	Coefficient	SE
**Total diet quality**
Nearest neighbor	−0.055	0.13
Radius matching (*r* = 0.1)	−0.002	0.09
Radius matching (*r* = 0.001)	−0.091	0.16
Kernel	−0.004	0.09
Stratification	−0.011	0.08
**BMI**
Nearest neighbor	−2.861^c^	0.95
Radius matching (*r* = 0.1)	−2.568^c^	0.57
Radius matching (*r* = 0.001)	−1.826^c^	1.08
Kernel	−2.631^c^	0.61
Stratification	−2.504^c^	0.72

*^a–c^Indicate significance at the 99, 95, and 90% level. A detailed description of the matching algorithms and their implementation in STATA following Becker and Ichino (45) can be found at www.stata-journal.com/sjpdf.html?articlenum=st0026*.

The results show that among the group of diabetes patients, nutrition supplement intake seems to serve as an indicator for individual’s heightened efforts to improve diet-health and manage body weight. The results for HEI confirm those presented in Table 3, in that individuals who report frequent consumption of nutrition supplements do not realize measurable improvements in total diet quality. We computed all HEI sub-scores and did not find any significant differences in diet quality for nutrition supplement takers among those diagnosed with diabetes.

Across matching algorithms, the results in Table 4 reject the hypothesis of a positive impact of nutrition supplements intake on diet quality for diabetes patients. However, we confirm significant and elevated differences in BMI status outcomes between diabetes patients who reported to take nutrition supplement and non-takers.

## Discussion

This study focuses on the investigating the linkage between diabetes, diet-health behavior, and health outcomes that are frequently discussed in the context of diabetes management, public health, and diet quality (as measured by the HEI-2010) and BMI. We use PSM as means of simulating a randomized control trial based on U.S. NHANES survey data to quantitatively test three hypotheses in the context of the importance of dietary adherence in management diabetes.

We determine (1) to what extent diabetes patients’ diet quality (HEI) outcomes differ compared to healthy individuals, and (2) whether patients’ improved and more appropriate dietary choices lead to lower BMI outcomes. The third objective and analytical step is motivated by dwindling levels of consuming fruits and vegetables and growing demand for nutrition supplements across Canada and the U.S. We hypothesized that the decision to frequently consume nutrition supplements could be associated with measurable positive effects on diet quality, thus leading to significantly lower BMI levels when contrasting supplement takers and non-takers. Our innovative analysis is based on the classic Grossman and Becker model, as we modeled the choice of nutrition supplement intake as an indicator for an active decision to invest into better health.

Our analysis reveals that being diagnosed with diabetes and likely having received dietary advice and nutrition guidelines do not necessarily translate into measurable improvements in total diet quality or its components based on our use of the HEI-2010 scoring system.

We contribute to the ongoing debates in the diabetes health community by addressing to what extent adherence to dietary guidelines and subsequent changes in patients’ diet-health behavior may lead to measurable and positive diet-health outcomes. Much of the discussion around dietary guidelines and consumer adherence has involved the socio-economic and demographic profiles of the population affected by diabetes. As such, variables such as age, gender, education, and income, previous studies have been frequently addressed. Our study shows that the risk of diabetes significantly increases with age and males are more likely to be diabetic. These effects are countered by increasing levels of educational attainment, often associated with better diet-health knowledge. Thus, actual diet behavior negatively affects the likely of being diagnosed with diabetes. Finally, higher household incomes (>$100,000), an antagonist to issues with food affordability, do not impact the probability of diabetes diagnosis.

We also investigate the linkage between diabetes and BMI. Diabetes and inactivity may lead to the metabolic syndrome, which includes a cluster of conditions that occur together, such as increased blood pressure, a high blood sugar level, excess body fat around the waist, and abnormal cholesterol levels. Metabolic syndrome is linked to insulin resistance, which may lead to diabetes when the body is unable to make enough insulin to keep the blood glucose within the normal range (49). We find that diabetes patients on average show lower BMI scores, leaving us to conclude that overall improvements in lifestyle management might stem from an individual changing exercise patterns or frequencies. These two lifestyle changes are an equally important component of living with diabetes. However, as is indicated by the selection model in Table 2, living an active lifestyle is among the strongest antagonists (by coefficient magnitude) to a diabetes diagnosis.

Our third objective focused on exploring whether a relevant and identifiable diet-health behavior – the decision to regularly consume nutrition supplements – could be associated with improved diet quality and/or diet health outcomes among the group of diabetes patients. We find that consumers who take vitamin supplements lead a healthy lifestyle, as displayed by lower levels of BMI. Thus, supplements may not serve as substitutes for healthy eating. Instead, vitamin supplements seem to complement an already-established healthy food consumption. Therefore, supplement intake may serve as a marker for healthy eating and healthy lifestyles, which indicates that the individual seems to care about his/her general well-being. The HEI diet quality score lies at 52 out of 100 for the average U.S. consumer and individual HEI component scores, such as for fruits and vegetables, at 40% of their recommended levels (15). Previous studies have argued that nutrition supplements may be needed especially for at-risk population groups, such as people with diabetes, in order to improve diet quality and health outcomes (32). In the context of diabetes, the economic affordability of recommended intake levels of fruits and vegetables has been discussed as potential barriers to meeting dietary guidelines by several studies (50).

Results indicate that the frequent intake of supplements among diabetes patients does not lead measurable improvements in diet quality. However, supplement takers do score significantly lower in terms of BMI, suggesting that heightened attention to health, as part of a diabetes appropriate lifestyle, is associated with a significantly better overall health score.

In the context of diabetes health, the consumption of foods recommended by dietary guidelines and other complementary means of maintaining a high quality of diet (e.g., through nutrition supplements) in combination with frequent physical exercise can be thought of as an investment in long-term overall health. Diet behavior is a key factor to managing diabetes and adhering to a recommended dietary regime has been documented to face a multitude of barriers (18–20).

Our study provides a unique contribution in diabetes health research. Understanding the relationship among factors that could promote or reduce adherence to the dietary guidelines may shed light on new ways for people with diabetes, their families, and their health care providers to support adherence to recommended dietary patterns and improve health outcomes. Results from this study may provide information for the creation of more effective diet-health education and diabetes policies, a topic that has spread far beyond the management of diabetes and nutrition science in Europe and North America.

## Conflict of Interest Statement

The authors declare that the research was conducted in the absence of any commercial or financial relationships that could be construed as a potential conflict of interest.
